# Long-Term Impact of HEAT Educational Intervention in the Emergency Department in Karachi, Pakistan

**DOI:** 10.5334/aogh.4749

**Published:** 2025-09-13

**Authors:** Uzma Rahim Khan, Syed Ghazanfar Saleem, Aliza Shah, Ahmed Raheem, Muskaan Abdul Qadir, Salima Kerai, Fozia Parveen, Saima Ali, Junaid A. Razzak, Nadeem Ullah Khan

**Affiliations:** 1 The Aga Khan University, Karachi, Pakistan; 2 Indus Hospital and Health Network, Karachi, Pakistan; 3 University of British Columbia, Vancouver, Canada; 4 Weill Cornell Medicine, New York, USA

**Keywords:** heat-related illnesses (HRIs), heatwave, emergency department (ED), educational training intervention, heat emergency awareness and treatment (HEAT), heat stroke, heat exhaustion, health system preparedness, emergency medicine, climate-resilient healthcare

## Abstract

*Background:* Karachi faced an unprecedented heatwave in 2015, causing severe health outcomes. The heat emergency awareness and treatment (HEAT) intervention was developed to train healthcare providers to identify and manage heat-related illnesses (HRIs). The HEAT intervention was implemented in major emergency departments (EDs) in Karachi in 2018.

*Objective:* This study evaluated the long-term impact of the HEAT intervention on ED physicians’ diagnosis and management of patients with HRIs in a single tertiary-care hospital.

*Method:* This study utilized time-series analyses to evaluate the long-term impact of HEAT intervention utilizing ten-year data (pre-intervention, 2013–2017 and post-intervention, 2018–2022). Data were obtained from a single hospital related to diagnoses and management of HRIs for the study period. The outcomes assessed were the number of HRIs diagnosed, use of intravenous (IV) fluids, and use of sponging and ice packs. A zero-inflated interrupted time series Poisson regression model was used to assess the impact of HEAT intervention on diagnosis and management of HRIs, while accounting for time and maximum ambient temperature.

*Findings:* At the crude level, analyses showed a decrease in the number of HRI diagnoses (estimate = −1.63, p < 0.001*), use of IV fluids (estimate = −0.72, p = 0.09), and in the use of sponging (estimate = −0.51, p = 0.64) in the post-intervention period. Findings from the sensitivity analyses, excluding the outlier observations due to the severe heat event of 2015, showed a statistically significant increase in HRI diagnoses (estimate = 2.18, p < 0.001*) and in the use of IV fluids (estimate = 2.07, p < 0.001*) in the post-intervention period.

*Conclusion:* Our educational training intervention was effective in improving HRI diagnosis and management among ED physicians from a select hospital over a long-term period. Findings need to be generalized with caution to other settings.

## Background

There has been an increase in the frequency and intensity of heat waves worldwide over the past decade [1], widely attributed to the ongoing climate crisis and rising greenhouse gas emissions [1, 2].

The city of Karachi, with a population of around 20 million people [3], is the largest in Pakistan. It experiences a semi-arid climate characterized by hot and humid summers and mild, dry winters. Due to its geographical location near the Arabian Sea, flat terrain, rapid urbanization, and rapid population growth, Karachi is particularly vulnerable to the effects of climate change, especially heat waves with temperatures exceeding 40°C [4, 5]. The deadliest heat wave in Karachi’s history occurred in 2015 and resulted in the tragic loss of approximately 1,200 lives, primarily among the elderly, poor, outdoor workers, and individuals with chronic ailments or disabilities [4]. Most of these fatalities were attributed to heat strokes and dehydration [4].

The city’s environmental challenge is compounded by its lack of trees, which contributes to increased greenhouse gas emissions and exacerbates the impacts of climate change [6]. In Karachi’s densely populated urban environment, studying exposure pathways during heat waves is essential to understanding their impact and designing targeted interventions. The city’s outdoor workers, such as street vendors, construction laborers, and rickshaw drivers, are particularly vulnerable to direct exposure, along with children, the elderly, and those with pre-existing health conditions [7]. High temperatures directly contribute to a surge in heat-related cardiovascular and respiratory illnesses, especially among these groups [8]. Indirect exposure pathways, such as Karachi’s chronic electricity shortages and erratic power outages, disrupt access to air conditioning and cooling systems, while the lack of adequate water supply further exacerbates dehydration risks [9, 10]. Many residents live in poorly ventilated homes, where the combination of high humidity, frequent load-shedding, and limited access to cooling intensifies heat emergencies. Hence, timely recognition of heat-related illnesses (HRIs) is important to mitigate the health impacts on Karachi’s vulnerable population. It has been shown that despite being aware of the health impacts of severe heat, trainees and healthcare providers do not feel adequately prepared to deal with the cases [11]. This can largely be attributed to the paucity of research on HRIs as well as the lack of algorithms and guidelines in hospitals. Presenting complaints of HRIs also overlap with local endemic and summer tropical infections. Our study builds on the heat emergency awareness and treatment (HEAT) intervention by Nadeem et al. [12] in four hospitals in Karachi in April 2018 to address the gap in knowledge and training of emergency medicine care providers regarding the diagnosis and management of HRIs in Pakistan [13] using a standardized treatment algorithm [10]. The analyses of HEAT intervention showed significant improvement in the diagnosis rate, temperature monitoring, as well as external cooling measures for patients presenting to emergency departments (EDs) with symptoms of HRIs [13]. Hence, we aim to evaluate the long-term adaptation of the HEAT intervention by comparing data on HRIs and management practices from the five-year pre (2013–2017) and post-intervention period (2018–2022) in a single hospital in Karachi, Pakistan.

## Approach

### Study design

This study utilized a retrospective, long-term time-series analysis to evaluate the impact of heat intervention on improvement in HRI diagnosis and management. It utilized hospital medical record data from a five-year pre-intervention (2013–2017) and a five-year post-intervention (2018–2023) period.

The HEAT intervention was implemented in April 2018. The intervention began with a scoping review of existing clinical practices and a qualitative study by Uzma et al. to gauge current perceptions and practices among ED healthcare providers [8]. This included teaching workshop, simulation scenario, pre- and post-test with MCQ assessment, and management in ED [12]. The intervention comprised five- to six-hour training and refresher workshops (refreshers done in July 2018), development of treatment algorithm, 13 heat emergency case simulated scenarios, and distribution of clinical practice manuals to equip healthcare providers with the skills needed to manage heat-related emergencies more effectively at all study sites for the ED. The original study employed a pre- and post-intervention design, collecting data prospectively in real time [12]. In contrast, the current study utilizes retrospective data to analyze the impact of the HEAT intervention.

The study setting is a non-governmental not-for-profit (welfare) hospital located in the Korangi district of Karachi, providing quality care at no cost to low-income communities in Karachi. The hospital’s ED has 24 beds and accommodates an average daily patient load of 500–550 individuals. The ED healthcare team consists of around 30 doctors, including Senior Medical Officers, Medical Officers, and 14 trainee residents. While the number of residents increased during the post-intervention period, the number of faculty and other healthcare providers remained unchanged.

The diagnosis recorded in the ED’s electronic medical record was based solely on the discretion and clinical judgment of the attending physician.

### Data source

The data were obtained from the electronic health record (EHR) system of the hospital. The variables extracted were patient demographics (age and gender), triage vitals (blood pressure, pulse rate, respiratory rate, and body temperature), number of heat diagnoses, temperature monitoring, water sponging, administration of intravenous (IV) fluids (sodium chloride and/or dextrose), mortality, length of ED stay, and disposition. Ambient temperature data for the months of May to July from 2013 to 2022 were obtained from the Pakistan Meteorological Department (PMD), corresponding to the same months for which patients’ medical records were extracted.

### Measures

The outcomes of the previous HEAT trial were frequency of diagnosis of HRIs (i.e., heat exhaustion, heat stroke) and management (such as temperature monitoring, use of external cooling measures, and IV fluids) by ED healthcare providers in patients presenting with potential and confirmed heat emergency conditions. For the purpose of consistency and comparability, we have utilized the same outcomes for the present study, i.e., number of heat-related diagnoses, number of IV fluid administrations, number of sponging, and use of ice packs at axilla and groin throughout the pre- and post-intervention periods. Given that we did not have data on temperature monitoring and removal of body clothing, these variables were not included in the present analyses. For temperature measures, the maximum daily ambient temperature and maximum average temperature were used.

### Statistical methods

Data were analyzed using SPSS version-21 (IBM Corp. Released 2012). Mean and standard deviation were reported for quantitative variables, while percentages and frequencies were calculated for categorical data. For data with non-normal distribution median and interquartile (IQR) were calculated. These methods were used to analyze socio-demographic data, clinical presentations, the number of heat-related diagnoses and management, and patient outcomes for the months of May, June, and July across pre- and post-intervention periods (2013–2022). Descriptive and bivariate analyses (using appropriate tests) were conducted to understand the crude relationships.

The main predictors in the model comprised time (as a continuous variable in days for the months of May, June, and July for the years 2013 till 2022), maximum daily ambient temperature (also continuous unit in degrees Celsius), and a binary variable representing the periods before and after the intervention (dichotomous). We used a time series regression analytic technique. A zero-inflated interrupted time series (ITS) Poisson regression was utilized to model the impact of heat intervention on the frequency of heat diagnosis, sponging, and IV fluid administration while accounting for time and maximum daily ambient temperature.

A sensitivity analysis was conducted and zero-inflated ITS Poisson regression was repeated by second excluding the 2015 heatwave days from June 20^th^ to July 1^st^, which is considered an outlier event in the pre-intervention period. It was considered an outlier because the heatwave was very extreme, and the number of cases were numerous and severe. Point estimate, standard errors, and 95% confidence intervals provided for each estimate are reported.

### Ethical considerations

Ethical review exemption was obtained from the Aga Khan University Ethical Review Committee and the Indus Health Network Institutional Review Board (IRB) given the nature of secondary analyses. The analyses were done on a de-identified dataset of patients. This study involved the use of retrospective data only, without any direct patient interaction. No patient identifiers were used, ensuring anonymity and confidentiality.

### Justification of the selected approach to assess the impact of adaptation responses on health outcomes for the case study

The hospital’s comprehensive EHR system provides retrospective records on patient diagnoses, treatments, and outcomes. This availability ensured that the necessary data for evaluating the intervention was readily accessible, significantly reducing the need for additional data collection efforts. This approach was especially aligned with the study’s requirements, as the guidelines explicitly prohibited the collection of primary data.

Additionally, all the faculty members involved in the initial intervention are still employed at the hospital, which offers a unique continuity in care and documentation practices. This consistency helps to minimize potential bias, particularly any variations in care practices that might arise due to faculty turnover.

## Results

A total of 308 patients diagnosed with HRI in the ED during May, June, and July from 2013 to 2022 were included in this study. Table 1 presents the socio-demographic analyses and clinical characteristics, comparing pre-intervention (2013–2017) to post-intervention (2018–2022) data.

**Table 1:** Socio-demographic and clinical characteristics of patients visiting the ED with heat-related illnesses pre-intervention (2013–2017) and post-intervention (2018–2022) (n = 308).

	PRE-INTERVENTION (2013–2017) (N = 184)	POST-INTERVENTION (2018–2022) (N = 124)	P-VALUE
Age in Years	51.43 ± 18.33	42.52 ± 17.39	<0.001*
Male	117 (64)	101 (82)	<0.001*
Female	67 (36)	23 (19)	
**Triage Category**			
Priority level 1	34 (19)	32 (26)	<0.043*
Priority level 2	49 (27)	37 (30)	
Priority level 3	94 (51)	45 (36)	
Priority level 4	7 (4)	10 (8)	
**Presenting Complain**			
Fever	135 (73)	71 (57)	<0.003*
Malaise/Nausea/Vomiting	70 (38)	110 (89)	<0.001*
Weakness	33 (18)	25 (20)	0.624
Altered Mental Status	26 (14)	26 (21)	0.116
Shortness of Breath (SOB)	13 (7)	26 (21)	<0.001*
Headache	10 (5)	24 (19)	<0.001*
Faint/Dizziness/Syncope	9 (5)	23 (19)	<0.001*
Fall	19 (10)	10 (8)	0.505
Chest	1 (1)	27 (22)	<0.001*
Abdominal Pain	2 (1)	22 (18)	<0.001*
Palpitations	8 (4)	15 (12)	<0.011*
Anxiety	17 (9)	6 (5)	0.150
Body ache	9 (5)	7 (6)	0.770
Chills	3 (2)	7 (6)	<0.051
Urinary	4 (2)	2 (2)	0.727
Gasping	1 (1)	4 (3)	0.068
Cramp	2 (1)	0 (0)	0.244
**Triage Vitals**			
Systolic Blood Pressure (BP) mmHg	117.03 ± 36.85	126.87 ± 23.83	0.009*
Diastolic Blood Pressure (BP) mmHg	70.59 ± 22.94	76.17 ± 14.53	0.017*
Heart Rate (HR)	101.07 ± 35.16	99.25 ± 26.45	0.624
Temperature (Celsius)	38.08 ± 4.45	37.55 ± 1.4	0.200

*p < 0.05 indicates statistical significance.

Table 1 reports socio-demographic and clinical characteristics of patients visiting the ED with HRIs pre-intervention (2013–2017) and post-intervention (2018–2022). There was a decrease in the average age of patients, from 51.43 to 42.52 years (p < 0.001). In the pre-intervention period, there were 117 male patients (64%) and 67 female patients (36%). Whereas, in the post-intervention period, males were 101 (82%) and females were 23 (19%) (p < 0.001).

The triage system used in the ED categorized patients into four priority levels based on clinical urgency: Priority 1 (P1) included patients with life-threatening conditions requiring immediate care; Priority 2 (P2) included serious but stable cases; Priority 3 (P3) covered non-critical but symptomatic individuals; and Priority 4 (P4) referred to minor or non-urgent cases. Among triage categories, there were more Priority 1 (P1) needing immediate attention in the post-intervention phase compared to the pre-intervention phase (19% vs 26%, p = 0.043). In the pre-intervention phase, the most common presenting complaints among patients with HRIs were fever (n = 135, 73%), malaise/nausea/vomiting (n = 70, 38%), and weakness (n = 33, 18%). Post-intervention, significant presenting complaints included malaise/nausea/vomiting (n = 110, 89%, p < 0.001), shortness of breath (n = 26, 21%, p < 0.001), headache (n = 24, 19%, p < 0.001), syncope (n = 23, 19%, p < 0.001), chest pain (n = 27, 22%, p < 0.001), and abdominal pain (n = 22, 18%, p < 0.001).

At triage, the systolic blood pressure (117 mmHg in pre-intervention vs 126.8 mmHg in post-intervention) (p = 0.009) and diastolic blood pressure (70.5 mmHg in pre-intervention vs 76.1 mmHg in post-intervention) (p = 0.017) were higher in post-intervention.

Table 2a reports crude differences in the number of heat-related diagnoses and management between the pre-intervention (2013–2017) and post-intervention periods (2018–2022). Diagnosis of heat exhaustion was greater in the n = 85 (69%) in post-intervention period (p < 0.001), as compared to the pre-intervention period n = 63 (34%), while heat stroke was reported more during pre-intervention n = 114 (62%) as compared to the post-intervention n = 29 (23%) (p < 0.001) (Table 2a).

**Table 2a:** Number of heat-related diagnoses and management pre-intervention (2013–2017) and post-intervention (2018–2022) (n = 308).

	PRE-INTERVENTION (2013–2017) N = 184 (%)	POST-INTERVENTION (2018–2022) N = 124 (%)	P-VALUE
**Heat Diagnosis**			
Total Heat Diagnosis	184 (59.7)	124 (40.3)	0.0008*
Heat Exhaustion	63 (34)	85 (69)	<0.001*
Heat Stroke	114 (62)	29 (23)	<0.001*
Heat Syncope	1 (1)	0 (0)	0.411
Heat Cramp	4 (2)	0 (0)	0.098
Heat Rashes	2 (1)	5 (4)	0.089
Heat intolerance	0 (0)	1 (1)	0.222
Any other heatrelated illness	0 (0)	4 (3)	0.014*
**Management**			
IV Fluid Administration	70 (39)	82 (66)	<0.001*
Sponging	42 (23)	17 (14)	0.046*
Ice Pack	2 (1)	2 (2)	0.689

*p < 0.05 indicates statistical significance.

Table 2b reports trends in the diagnosis of HRIs pre-intervention (2013–2017) and post-intervention periods. Heat exhaustion cases were minimal throughout the pre-intervention period with n = 0 in 2013 and n = 1 in both 2014 and 2016. Heat stroke cases were more prevalent, peaking at n = 108 in 2015, while other HRIs were infrequent. Whereas, heat exhaustion cases were reported more during post-intervention, reaching n = 31 in 2018 and fluctuating in subsequent years, with n = 23 in 2019, n = 8 in 2020, n = 14 in 2021, and n = 9 in 2022. Heat stroke cases were fewer in the post-intervention period.

**Table 2b:** Yearly trend in the diagnosis of heat-related illnesses pre-intervention (2013–2017) and post-intervention (2018–2022).

YEAR	HEATH EXHAUSTION 148 (%)	HEAT STROKE 143 (%)	OTHER HEAT-RELATED ILLNESSES* 4 (%)
**Pre-Intervention**			
2013	0 (0%)	1 (1%)	0 (0%)
2014	1 (1%)	1 (1%)	0 (0%)
2015	58 (39%)	108 (76%)	5 (29.5%)
2016	1 (1%)	1 (1%)	1 (5.9%)
2017	3 (2%)	3 (2%)	1 (5.9%)
**Post-Intervention**			
2018	31 (21%)	5 (3%)	4 (23.5%)
2019	23 (16%)	9 (6%)	1 (5.9%)
2020	8 (5%)	2 (1%)	1 (5.9%)
2021	14 (9%)	9 (6%)	4 (23.5%)
2022	9 (6%)	4 (3%)	0 (0%)

*Other HRIs include heat syncope, heat cramps, heat rashes, heat intolerance, and any other heat-related illness.

**Figure 1:**
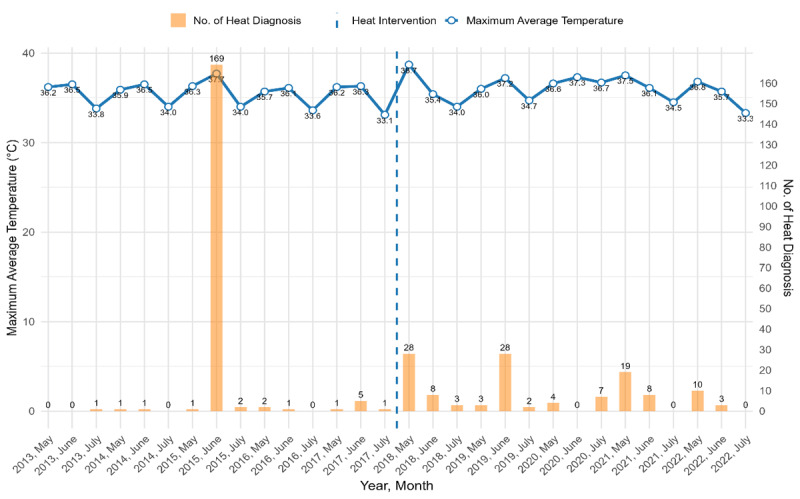
Trends in heat-related diagnoses and their correlation with maximum average ambient temperature in summer months (May, June, and July) pre-intervention (2013–2017) and post-intervention (2018–2022).

Figure 1 illustrates the trend of heat-related diagnoses for May, June, and July between 2013 and 2022. The line shows the maximum average temperature over the years. The bars represent the number of heat-related diagnoses. The highest spike occurred in June 2015, with a peak of 169 cases with maximum average ambient temperature of 37.7°C. Following 2015, the number of heat diagnoses stayed low throughout the pre-intervention years (2013, 2014, 2016, and 2017). In the post-intervention (2018–2022), despite some fluctuations, the trend shows an increased number of diagnoses. There is an observed relationship between heat diagnoses and maximum average temperature; diagnoses tend to go up on high average temperature days.

**Figure 2:**
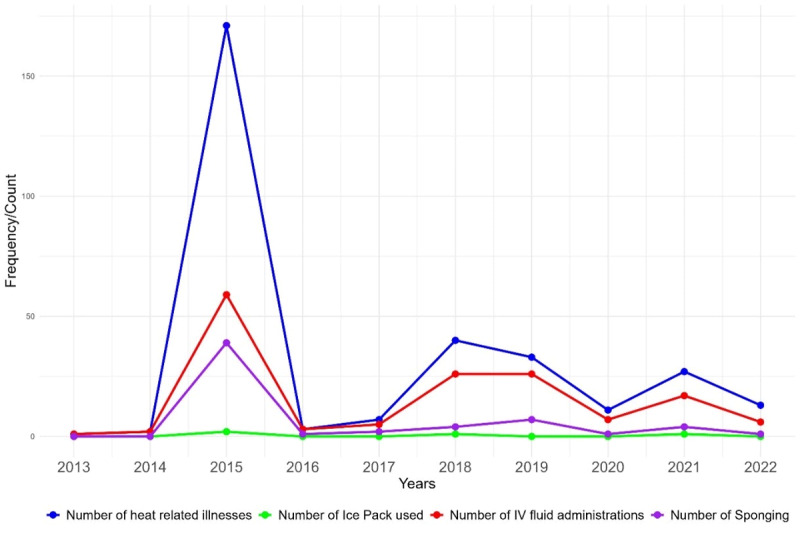
Yearly trends in diagnosis and management of heat-related illnesses pre-intervention (2013–2017) and post-intervention (2018–2022).

Figure 2 illustrates trends in the diagnosis and management of HRIs, focusing on the use of IV fluids, sponging, and ice packs before and after a specified intervention. In 2015, there was a notable peak in HRIs, with less than half receiving IV fluids, even a smaller number receiving sponging, and only a few treated with ice packs. Following the intervention, there was a consistent increase in the administration of IV fluids as heat diagnoses increased each year. The use of sponging also showed an upward trend, with cases increasing from 2018 to 2019. Although the gap between heat diagnoses and IV fluid administration narrowed in the post-intervention period, the gap for sponging remained unchanged.

Table 3 reports differences in patient outcomes for HRIs between pre-intervention (2013–2017) and post-intervention periods (2018–2022). In the pre-intervention period, out of 184 patients, n = 94 (51%) were discharged, n = 36 (20%) were referred to other hospitals, n = 49 (27%) were admitted, and n = 5 (3%) died (p < 0.001). In the post-intervention period, among 124 patients, n = 82 (66%) were discharged, n = 25 (20%) were referred to other hospitals, n = 4 (3%) were admitted, and n = 1 (1%) died (p < 0.001) (Table 3).

**Table 3:** Patient outcomes for heat-related illnesses pre-intervention (2013–2017) and post-intervention (2018–2022) (n = 308).

	PRE-INTERVENTION (2013–2017) (N = 184)	POST-INTERVENTION (2018–2022) (N = 124)	P-VALUE
**ED Disposition**			
Discharged	94 (51)	82 (66)	<0.001*
Referral	36 (20)	25 (20)	
Leave against medical advice (LAMA)	0 (0)	12 (10)	
Admission	49 (27)	4 (3)	
Expired	5 (3)	1 (1)	
**Admissions**	**(n = 49)**	**(n = 4)**	
HDU/CCU/ICU	16 (33)	3 (75)	0.089
General Ward Adults	33 (67)	1 (25)	
**Length of Stay (Median IQR in minutes)**	148 (1440 - 38)	245 (383 - 147)	<0.001*
**Mortality**			
ED Mortality	5 (3)	1 (1)	0.234
Hospital Mortality (n = 53)	11 (6)	2 (2)	0.062
Total Mortality	16 (9)	3 (2)	0.025*

HDU/CCU/ICU*= High Dependency Unit, Critical Care Unit, Intensive Care Unit.

Moreover, out of 53 admitted patients, n = 16 (33%) required high dependency or critical care units (HDU/CCU/ICU) in the pre-intervention period, while n = 3 (75%) required critical care in the post-intervention period (p = 0.089). Admissions to the general ward remained at n = 33 (67%) in the pre-intervention and n = 1 (25%) post-intervention. The Length of Stay (LOS) was 148 minutes (range: 38–1440) during the pre-intervention period, while it was 245 minutes (range: 147–383) during the post-intervention (p < 0.001). Total mortality decreased from 9% to 2% (p = 0.025) during pre- and post- intervention periods.

### The interrupted time series (ITS) analyses

The ITS analyses (Table 4a), utilizing zero-inflated Poisson regression on data from 2013 to 2022 for the months of May to July, showed a reduction in heat-related diagnoses in the post-intervention phase (estimate = −1.63, p < 0.001*). However, this reduction was influenced by a particularly high number of cases in a single year, i.e., 2015, during the pre-intervention period, which may have affected the overall findings. Similarly, a statistically insignificant reduction in IV fluid administrations (estimate = −0.72, p = 0.09) and in sponging usage (estimate = −0.51, p = 0.64) was observed in the post-intervention period.

**Table 4a:** Interrupted time series (ITS) analyses from 2013 to 2022 for the months of May to July.

	ESTIMATE (β)	P-VALUE	CI (95%)
**Heat Diagnosis**	–1.63	<0.001*	–2.28 to −0.99
Heat Exhaustion	−0.74	0.34	−2.29 to 0.80
Heat Stroke	−4.06	0.056	−8.2 to 0.10
**IV Fluids**	–0.72	0.09	–1.5 to 0.13
**Sponging Use**	–0.51	0.64	–2.70 to 1.68

*Ice pack was not included in this table due to the limited cell count.

**Sensitivity analyses:** The above regression analyses were repeated, excluding the data points in the extreme heatwave year (June 15th to July 1st) which was considered an outlier (Table 4b). The results showed a significant increase in heat diagnoses during the post-intervention phase (estimate = 2.18, p =<0.001*). Similarly, there was a significant increasing trend in IV fluid administrations (estimate = 2.07, p =<0.001*) and an insignificant increase in sponging usage (estimate = 1.82, p = 0.23) during the post-intervention phase.

**Table 4b:** Analyses from 2013 to 2022 for the months of May to July excluding the extreme heat days in 2015.

	ESTIMATE (β)	P-VALUE	CI (95%)
**Heat Diagnosis**	2.18	<0.001*	1.21 to 3.16
Heat Exhaustion	0.45	0.014*	0.15 to 0.98
Heat Stroke	0.44	0.081	−0.95 to 0.58
**IV Fluids**	2.07	<0.001*	1.08 to 3.25
**Sponging Use**	1.82	0.23	–1.16 to 4.82

*Ice pack was not included in this table due to the limited cell count.

## Discussion

This study examined the effect of a hospital-based heat management training program by comparing five years of data before and after the intervention. The crude analysis showed a significant decline in total heat-related diagnoses in the post-intervention period, accompanied by a marked shift in diagnostic patterns. Heat exhaustion became the most common diagnosis, while heat stroke declined sharply. Management practices also evolved, with IV fluid use increased significantly, while sponging declined and ice pack use remained rare.

When trends over time were explored using ITS analysis, there was an overall decline in reported heat diagnoses after training, with heat stroke cases showing a borderline significant decrease. However, changes in exhaustion, IV fluid administration, and sponging were not significant in this model, indicating that crude declines may partly reflect underlying year-to-year variability. Because the 2015 heatwave was an exceptional event, coinciding with Ramadan fasting, prolonged electricity breakdowns, and intense media attention due to high mortality, it was considered separately. Once this outlier period was excluded, the sensitivity analysis revealed a different picture: total heat diagnoses, exhaustion, and IV fluid use showed significant increases in the post-intervention years, while stroke and sponging showed only small, non-significant increases. This reversal indicates that the apparent decline in crude analyses was largely driven by the extraordinary burden of 2015. A study conducted on mortality data from the 2015 heatwave period in Karachi found that residents were approximately 17 times more likely to die from heat-related causes in June 2015 compared to June 2014. The analysis, based on manual review of death certificates, reported the severe impact of the extreme heat event [14]. The unusually high number of heat stroke diagnoses during that year may represent not only a true surge in severe cases but also possible misclassification of heat exhaustion as stroke, reflecting limited diagnostic experience at the time.

Taken together, the results suggest that the training may not have reduced the absolute number of HRIs but likely contributed to improved diagnostic accuracy and more consistent clinical management. The sharp decline in heat stroke diagnoses alongside the rise in exhaustion points to earlier recognition of milder illness, potentially preventing progression to severe heat stroke and reducing misclassification. The increased and consistent use of IV fluids further supports the interpretation that staff became more confident in applying appropriate management protocols. The persistence of some heat stroke cases in the post-intervention period, although fewer underscores that severe illness remains a risk. Fasting during Ramadan is likely an important driver, as dehydration and reduced heat tolerance make individuals more susceptible to heat stroke, even when preventive and clinical measures are in place. Our observation of an increase in heat exhaustion diagnoses post-intervention, alongside a reduction in heat stroke, aligns with findings from interventional studies in ED settings. The Heat Alert Pathway, developed for EDs and pre-hospital providers, emphasizes early recognition of heat illness and protocolized management steps, including rapid cooling, IV fluid resuscitation, and close monitoring. A study highlighted that structured training and stepwise algorithms shifted clinicians toward earlier categorization of milder heat illness, thereby preventing progression to severe heat stroke and improving management fidelity [15]. Similarly, the U.S. Department of Defense Clinical Practice Guideline for Heat Illness (2024) stresses that standardized provider education and adherence to treatment protocols facilitate recognition of exertional heat illness before Central Nervous System (CNS) dysfunction occurs, which reduces misclassification and promotes consistent use of cooling and fluids [16]. This aligns with previous research demonstrating that standardized treatment guidelines and protocols enhance healthcare providers’ ability to initiate timely and effective management of chronic conditions such as hypertension, diabetes mellitus, and cardiovascular disease as well as certain acute emergencies [17, 18].

A key strength of this study is the use of the same months, May to July, for both periods, reducing seasonal bias. Nonetheless, year-to-year variation in daily temperature, humidity, and heat index could not be controlled and may partly explain fluctuations in case numbers. Another important factor is faculty stability in the study hospital, which strengthens internal validity by ensuring that the same clinicians were exposed to both the 2015 crisis and subsequent training. This combination may have amplified the long-term impact of the intervention. At the same time, it limits generalizability, as hospitals without stable staff or structured residency programs may not experience similar improvements.

## Conclusion

The study indicates that the intervention had a notable long-term effect on heat-related diagnoses with increased heat exhaustion diagnoses and the administration of IV fluids. Extending similar training to other hospitals, particularly those with high staff turnover, could provide a clearer picture of its impact and strengthen health system preparedness and resilience against future heatwaves. Additionally, the results may be more relevant to teaching hospitals and might not be directly generalized to other healthcare settings or institutions.
